# The Health Literacy Questionnaire: Initial Validity Testing in a Norwegian Sample

**DOI:** 10.3928/24748307-20200903-01

**Published:** 2020-10-08

**Authors:** Kristin Hjorthaug Urstad, Randi Andenaes, Astrid K. Wahl, Lisbeth G. Kvarme, Sølvi Helseth, Torbjørn Moum

## Abstract

**Background:**

The Health Literacy Questionnaire (HLQ) is a multidimensional generic questionnaire developed to capture a wide range of health literacy needs. There is a need for validation evidence for the Norwegian version of the HLQ (N-HLQ).

**Objective:**

The present study tested an initial version of the Norwegian HLQ by exploring its utility and construct validity among a group of nursing students.

**Methods:**

A pre-test survey was performed in participants (*N* = 18) who were asked to consider every item in the N-HLQ (44 items across nine scales). The N-HLQ was then administered to 368 respondents. Scale consistency was identified and extracted in a series of factor analyses (principal component analysis [PCA] with oblimin rotation) demanding a nine-dimension solution performed on randomly drawn 50% of the samples obtained by bootstrapping. Correlations between the nine factors obtained in the 13-factor PCA and the scale scores computed by the scale scoring syntaxes provided by the authors of the original HLQ were estimated.

**Key Results:**

The pre-test survey did not result in the need to rephrase items. The internal consistency of the nine HLQ scales was high, ranging from 0.81 to 0.72. The best fit for reproduction of the scales from the original HLQ was found for these dimensions: “1. feeling understood and supported by health care providers,” “2. having sufficient information to manage my health,” and “3. actively managing my health.” For the dimensions “7. navigating in the healthcare system” and “8. ability to find good health information,” a rather high degree of overlap was found, as indicated by relatively low differences between mean highest correlations and mean next-highest correlations.

**Conclusions:**

Despite some possible overlap between dimensions 7 and 8, the N-HLQ appeared relatively robust. Thus, this study's results contribute to the evidence validation base for the N-HLQ in Norwegian populations. **[*HLRP: Health Literacy Research and Practice*. 2020;4(4):e190–e199.]**

**Plain Language Summary:**

This study tested the Norwegian version of the Health Literacy Questionnaire. The questionnaire (44 items across nine scales) was completed by 368 nursing students. Despite some overlap between scale 7 (“navigating in the health care system”) and scale 8 (“ability to find good health information”), the questionnaire appears to serve as a good measurement for health literacy in the Norwegian population.

People are increasingly given responsibility for own their health and, consequently, health literacy (HL) has become a topic of growing interest. Research has demonstrated associations between low functional HL (i.e., health-related reading and numeracy ability) and poor health-related outcomes, such as increased hospital admissions and readmissions (Baker et al., 2002; Chen et al., 2013; Choet et al., 2008; Scott et al., 2002), less participation in preventive activities (Adams et al., 2013; Thomson & Hoffman-Goetz, 2012; von Wagner et al., 2007), poorer self-management of chronic conditions (Aung et al., 2012; Gazmararian et al., 2003; Williams et al., 1998), poorer disease outcomes (Peterson et al., 2011; Schillinger et al., 2002; Yamashita & Kart, 2011), lower functional status (Wolf et al., 2005), and increased mortality (Bostock & Steptoe, 2012; Peterson et al., 2009; Sudore et al., 2006). More recently, using more dynamic and multidimensional measures of HL, associations have also been found with screening behavior, diabetes control, and patients' perceptions of quality of life (Jovanić et al., 2018; O'Hara et al., 2018; Olesen et al., 2017).

HL is defined in numerous ways. However, the current common understanding is that HL is a multidimensional concept comprising a range of cognitive, affective, social, and personal skills and attributes. According to Nutbeam (2008), HL contains three levels, progressing from basic skills in reading and writing (functional HL) to the ability to derive meaning from different forms of communication and apply new information to changing situations (interactive HL) and finally the ability to achieve policy and organizational changes (critical HL).

Although research suggests unambiguous associations between HL and health outcomes, a major shortcoming of such findings is that data have frequently been derived from suboptimal instruments (Jordan et al., 2011). Jordan et al. (2011) have highlighted limitations regarding the general conceptualisation of HL, coupled with weak psychometric properties of the instruments used, and they claimed that HL has not been consistently measured, thus making it difficult to interpret and compare HL at the individual and population levels.

Until recently, most instruments measuring HL have been unidimensional, focusing on health-related numeracy and reading skills (Berkman et al., 2010). For example, instruments frequently used to measure HL include the Rapid Estimate of Adult Literacy in Medicine focusing on word recognition (Davis et al., 1993) and the Test of Functional Health Literacy (Parker et al., 1995) testing health-related reading and numeracy skills. Similarly, the Newest Vital Sign is a commonly used instrument focused on testing a respondent's understanding of a food label (Weiss et al., 2005). Therefore, a need for more comprehensive assessment tools has recently been recognized (Altin et al., 2014), and in the past 5 to 6 years multidimensional assessment tools have been developed, such as the European Health Literacy Survey Questionnaire (HLS-EU-Q) (Sørensen et al., 2013) and the Health Literacy Questionnaire (HLQ) (Osborne et al., 2013), yielding a more multifaceted picture of HL. Although the HLS-EU-Q was developed and tested among the Norwegian population (Finbråten et al., 2017; Sorensen et al., 2013), there is a need for additional multidimensional HL instruments in the Norwegian setting, such as the HLQ. The HLS-EU-Q was specifically designed to compare populations using three dimensions; however, the advantages of the nine-dimension HLQ questionnaire is that it can provide a nuanced evaluation of education programs and derive HL profiles that might, in turn, facilitate intervention development and service improvement (Batterham et al., 2014). The HLQ has been translated into more than 15 languages. It has undergone validity testing in Germany, Denmark, France, and Slovakia and has been shown to have strong psychometric properties in the translated versions as well as the original (Debessue et al., 2018; Elsworth et al., 2016; Kolarčik et al, 2015; Maindal et al., 2016; Nolte et al., 2017). In the last couple of years, the HLQ has also been used in Norwegian settings (Larsen et al., 2018; Stømer et al., 2019). Therefore, it is important to explore the utility and construct validity of the Norwegian version of the HLQ.

There is a growing acceptance of the view that the validation of self-reported instruments should be seen as an accumulation and evaluation of sources of validity evidence (American Psychological Association & National Council on Measurement in Education, 2014; Kane, 2013; Zumbo & Chan, 2014). Rather than relying on a strict factor analysis of psychometrical properties alone, according to Hawkins et al., (2018), the validation of HLQ should be based on a network of different empirical evidence that supports the intended interpretation and use of HLQ scores. As such, using a student sample for the initial validation of the questionnaire would contribute to the basis of validation evidence, especially in the context of a younger population. Hence, the current study aims to implement and test an initial version of the Norwegian HLQ, exploring its utility in the field as well as testing its construct validity within a group of nursing students.

## Method

### The Health Literacy Questionnaire

The HLQ is based on the World Health Organization's (WHO) definition of HL, described as the “cognitive and social skills which determine the motivation and ability of people to access, understand and use information in ways which promote and maintain good health” (Nutbeam, 1998, p. 13). The three levels of health literacy described in Nutbeam's (2008) theoretical model (i.e., functional HL, interactive HL, and critical HL) are incorporated within and across the domains of HLQ, thereby providing the possibilities to capture respondents' capability at each of these levels (Osborne et al., 2013). This link between Nutbeam's schema of HL and the HLQ was revealed through a validity-driven item-writing process based on citizens' lived experiences (Osborne et al, 2013), as illustrated in **Table 1**.

The development process of the HLQ consisted of two concept mapping workshops involving 27 workshop participants, comprising a patient group (*N* = 12) and a health care professional and researcher group (*N* = 15). Due to the concept mapping process developed by Trochim et al., (1994), a structured brainstorming process was initiated. The workshop participants were introduced to seeding statements based on the WHO's definition of HL, such as “thinking broadly about your experiences in trying to look after your health, what abilities does a person need to have to get, understand, and use health information to make informed decisions about their health?” The process confirmed that health literacy encompasses a broad range of concepts (Buchbinder et al., 2013; Jordan et al., 2011).

The next step of the development process included interviews with community members and patients and extensive validity testing in a construction sample (*N* = 634) and a confirmation sample (*N* = 405) in Australia (Osborne et al., 2013). The HLQ consists of 44 items in nine domains of health literacy. The first 5 scales, constituting part 1 of the HLQ, are scored on a 4-point, Likert-type response scale (*strongly disagree, disagree, agree, strongly agree*). The last four scales, constituting part 2, are scored on a 5-point response scale, where respondents rate the item levels by the difficulty in undertaking a task (*cannot do, usually difficult, sometimes difficult, usually easy, always easy*). The HLQ scales are listed in **Table 2**. The 44 items are published in the original HLQ validation paper (Osborne et al., 2013).

***Translation process.*** The translation and cultural adaptation of the HLQ into Norwegian followed a standardized protocol provided by the authors of the HLQ (Hawkins et al., 2020). Forward translation was guided by comprehensive item intent descriptions, and items were then reviewed for cultural appropriateness and measurement (linguistic) equivalence. Blind-back translation was also undertaken. The meaning of every nuance in the final translation was verified with one of the authors of the orignal HLQ (Osborne et al., 2013) through two consensus conferences in addition to written reports.

We performed a web survey pre-test to capture how the wording of the translated items were understood and experienced by Norwegian responders. Participants were included by a convenient sampling method, and the 18 participants (10 women, 8 men) were all citizens who lived in the western part of Norway, ranging in age from 30 to 80 years. The participants were given access to the web-based HLQ after giving their consent to participate. After finishing the questionnaire, participants were asked to consider every item in terms of (1) difficult to answer, (2) unclear, (3) use of difficult words, or (4) upsetting. Participants were also asked to provide comments or suggest alternative words or terms in a free-text response alternative at the end of the survey.

***Setting.*** The sample method used for testing the structure validity of HLQ was a convenience sample collected at two different universities in Norway in 2016: one in the capital of Norway, with the largest number of nursing students in the country, and the other in western Norway, the third most densely populated urban area in Norway. The population invited was nursing students in the first semester of the bachelor's degree program. The number of first-semester nursing students in 2016 was 302 and 570 for the two universities, respectively, and the grade point average requirement for admission to the two nurse education programs was 4.6 and 4.7 (grading range: 2 to 6).

Permission to conduct the study was obtained from both universities. Information about the study, including the purpose, scope, content, confidentiality, voluntary nature of participation, and the ability to withdraw from the study at any time, was provided to students in both oral and written formats.

A web-based questionnaire was made available on the students' official learning platform. Students who agreed to participate answered the questionnaire on campus after a lecture. The link to the questionnaire was available for about 40 to 60 minutes, and the link was closed after the last student finished the questionnaire. It was only possible for the participants to answer the questionnaire on campus. Therefore, no personal internet protocol addresses were collected or stored for the web-based survey. No information about personal or health issues was collected; thus, according to Norwegian law, the study did not require formal ethical approval from the Norwegian National Ethics Committee.

***Respondents.*** At the capital university, the recruitment of participants was performed after lectures provided for three different student groups. Of 181 nursing students attending the group lectures, 147 agreed to participate. At the university in western Norway, the data collection was performed after a lecture provided for the total group of first-semester nursing students. Here, a total of 250 were invited, and 221 agreed to participate. Thus, the total population of first-semester nursing students at the two universities was 872 and, of the 432 invited to participate, 368 agreed (85% response rate).

Respondents' ages ranged from 18 to 64 years, and the mean age was 24 years. Most of the sample were women (86%) and who were living with someone (76%). Furthermore, 83% of the sample spoke Norwegian when growing up, and 18% reported having a long-term health problem or disease. Participants' background characteristics are summarized in **Table 2**.

## Analyses

**Figure 1.**
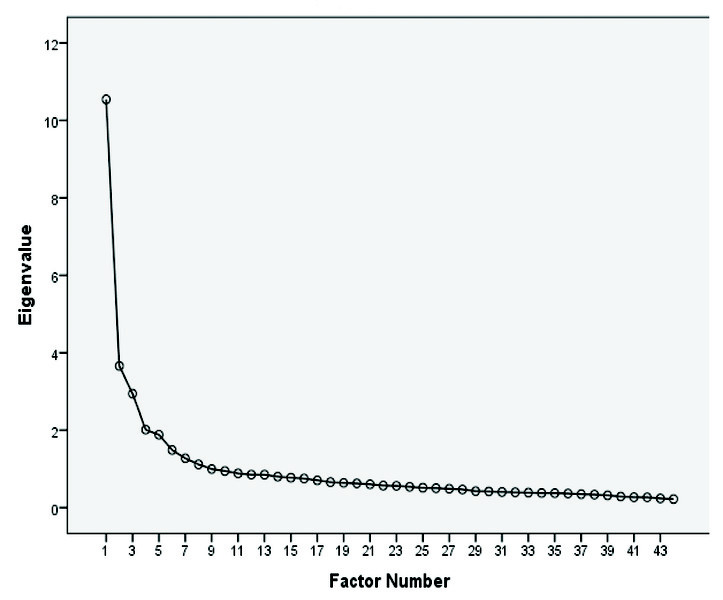
Scree plot for the 44-item Health Literacy Questionnaire.

The HLQ scales were calculated by adding the item scores and dividing the number of items in each respective scale; the first 5 scales ranged between 1 and 4 while the remainder ranged between 1 and 5 (Osborne et al., 2013) using SPSS Statistics version 22. The internal consistency of each of the predefined HLQ scales was assessed using Cronbach's alpha. We initially performed exploratory factor analyses and confirmatory analyses. Screen plots from exploratory factor analyses gave no clear indications as to the number of dimensions to be extracted, and a confirmatory analysis of the nine dimensions yielded an unsatisfactory fit (**Figure 1**).

To explore if a subset of the nine dimensions proposed by Osborne et al. (2013) could be consistently identified and extracted, a series of 13 factor analyses (principal component analysis [PCA] with oblimin rotation) demanding a nine-dimension solution was performed on randomly drawn 50% of the samples, obtained by bootstrapping. The purpose of choosing bootstrapping was that it would give us an understanding of how stable our results from the PCA were when repeating the analysis on randomly drawn samples. In this study, the analysis was thus repeated 13 times. Bootstrapping is considered a valuable resampling methodology as it can provide a large number of permutations of the sample, thereby enabling us to analyze samples and provide summary statistics (Costello & Osborne, 2005). The advantages of bootstrapping are that it does not require normal distribution—even with limited sample size, and it allows researchers to move beyond traditional parameter estimates to any statistic estimates, such as structure and pattern coefficients (Lu et al., 2014; Zientek & Thompson, 2007).

To compare results from the 13 rounds of factor analyses performed on these randomly drawn subsamples, factor scores were retained as separate variables (“factor scores”) for each round. Correlations between the nine factor scores obtained in the 13 nine-factor PCA and the a priori scales provided by Osborne et al. (2013) could then be estimated, thereby enabling us to assess which of the nine scales proposed by Osborne et al. (2013) could actually be retrieved with some consistency in our own sample.

## Results

In the web-survey pre-test, participants commented that the following items in the questionnaire appeared to be unclear from their perspective: item 7: “*When I see new information about health, I check up on whether it is true or not”* (reported as unclear by *n* = 3), item 8 “*I have at least one health care provider I can discuss my health problems with”* (reported unclear by *n* = 4), item 11 “*If I need help, I have plenty of people I can rely on”* (reported as unclear by *n* = 1), and item 22 “*I can rely on at least one healthcare provider”* (reported as unclear by *n* = 1). Participants suggested no concrete alternatives for these items. No participants reported that any of the items were “difficult to answer,” had “difficult words,” or were “upsetting.”. Suggested alternative wordings brought up during in-depth discussions were found to put the Norwegian version at risk of deviating in meaning from the original English version. Therefore, discussions did not result in rephrasing of any of the items.

## Scalability/Internal Consistency

Overall, the internal consistency of the nine HLQ scales was high, ranging from 0.81 to 0.72 (**Table 3**). The highest internal consistencies were found for “1. Feeling understood and supported by health care providers” (0.81) and “6. Ability to actively engage with healthcare providers” (0.81). The lowest were for the scales “9. Understanding health information well enough to know what to do” (0.72) and “5. Ability to find good health information” (0.72). Thus, homogeneity within scales measured by internal consistency was satisfactory for all subscales.

## Dimensional Structure

For each of the 13 separate nine-dimensional factor analyses (PCA) performed among randomly selected subsamples, the factors most closely reflecting the original nine scales were identified, allowing us to correlate factor scores with the scale scores derived from the a priori scales. High correlation indicated that the PCA had achieved a close correspondence with the original scales, thereby confirming the a priori nine-dimensional structure. Consistently high correlations between the *a priori* scale scores and the factor scores indicate a good fit, and the large difference between the highest and the next-highest correlation obtained in each of the 13 trials provides further evidence of the distinctiveness of the factors obtained. The highest average correlation and the difference between mean highest correlation and mean next-highest correlation of the nine factor scores and the HLQ index syntax score ranged from 0.950 to 0.686 and 0.157 to 0.622, respectively (**Table 2**).The best fit or reproduction of the scales from the original HLQ were found for “1. Feeling understood and supported by health care providers”, “2. Having sufficient information to manage my health,” “3. Actively managing my health,” and “5. Appraisal of health information.” The scales “7. Navigating in the health care system” and “8. Ability to find good health information” showed a rather high degree of overlap, as indicated by relatively low differences between mean highest correlations and mean next-highest correlations.

## Discussion

The purpose of this study was to acquire initial insights into the construct validity of the HLQ. Our validity testing indicated that the questionnaire seemed relatively robust as the items showed acceptability by respondents and the scales shows overall good internal consistency. The nine factors extracted were relatively distinct, thereby making it possible, in principle, to compare scale scores across populations.

According to our results, scales “7. Navigating the health care system” and “8. Ability to find good health information” in part 2 of the N-HLQ were the hardest to recapture. This finding is supported by previous HLQ validation reports. An overlap of scales 7 to 9 was reported in the Danish HLQ validation study (Maindal et al., 2016), and strong associations among scales 7 to 9 were noted in the German HLQ validation study (Nolte et al., 2017) as well as the original English version (Osborne et al., 2013). In the present study, scale “9. Understanding health information well enough to know what to do” was found to be the next weakest factor after scales 7 and 8. The inter-factor correlations between these scales might be characterized as medium-sized, with correlation between 7 and 8, 7 and 9, and 8 and 9, being 0.59, 0.56, and 0.70, respectively. Stronger associations between these factors were found in the German validation study (characterized as large inter-factor correlations; Nolte et al., 2017). The authors of the Danish validation study (Maindal et al., 2016) argued that the reason for the overlap among scales 7 to 9 might be a high-order factor or causal linkages determining the stronger associations among them. Scales 8 and 9 focus on the ability to locate (scale 8) and appraise (scale 9) health information, whereas scale 7 (health system navigation) may be seen as being a closely linked outcome of these abilities (Maindal et al., 2016). Our data support these previous findings.

## Study Limitations

A limitation of the current study might be that it is based on a relatively young and homogeneous group of participants with limited experience with the health services. Furthermore, the participants were bachelor degree-seeking nursing students, and the health perspective of their studies might somehow affect their perceptions of health care and their responses. However, including students for validation of self-reported instruments is regarded as an acceptable strategy in social behavioral and health sciences (van Ballegooijen et al., 2016). To reduce the influence of the participants' educational background in the current study, we included only first-semester students as they were at the beginning of their education process. We also support the view of Hawkins et al. (2018), who claimed that the validation of HLQ should be based on a range of validation evidence; thus, the current study contributes to the basis of validation evidence, especially in the context of a younger population. However, as HLQ is designed to be implemented across populations, further work with a wider range of respondents, including community health, hospital, and home care settings, is needed.

Compared to test-based tools such as Rapid Estimate of Adult Literacy (Davis et al., 1993) and the Test of Functional Health Literacy (Parker et al., 1995), which have more objective outcomes, there might a risk that measures from self-reported instruments such as HLQ are biased (Chang et al., 2019). Biased self-reported estimates can occur for various reasons, including gender differences. (Ruiz-Cantero et al., 2007; Weber at al., 2019). However, in the development phase of the HLQ, items biased for gender were eliminated (Osborne et al., 2013). Furthermore, several previous HLQ studies have looked at whether gender is a bias and concluded that it was not a threat to the results (Aaby et al., 2017; Friis et al., 2016).

Due to differences across cultures, the translations and adaptions of questionnaires regarding health and health care may be challenging (Acquadro et al., 2008; Epstein et al., 2015). Literature reviews have not yet revealed any gold standard procedure for such processes. However, the inclusion of an expert panel and back-translations seem to be essential elements (Epstein et al., 2015). The translation and adaption process in our study followed the process recommended by the authors of the original questionnaire, who included both of these steps (Hawkins et al., 2018). The value of including an expert panel with a clinical health background should be highlighted. In line with the German validation study, the translation processes included a discussion of some of the health terms in the questionnaire, such as health care providers (Nolte et al., 2017). Although the authors of the German validation study adjusted this to “doctors and therapists,” the consensus was to choose a term that was close to the original for the Norwegian HLQ. The pilot testing in this study did not indicate that respondents had any difficulties comprehending the chosen term.

Despite the indication that the pre-test included some uncertainty about four of the items, in-depth discussions did not result in any revisions. Finding the balance between a linguistic and conceptual equivalence to the original questionnaire is not a straightforward process (Chang et al., 1999). The translation team followed a structured translation procedure that included several extensive discussions of the items during which both cultural and linguistic aspects were considered. Thus, we trusted that the final version would be appropriate.

At this stage, there is scant evidence of the predictive validity of the HLQ. However, it is important to generate evidence of the HLQ's performance in cross-sectional studies and various populations at step one. As many studies have shown (Debussche et al., 2018; Kolarčik et al., 2015; Maindal et al., 2016; Nolte et al., 2017), the HLQ has acceptable strong properties in diverse settings, and future research should demonstrate whether the questionnaire predicts important outcomes such as improved access to services, better use of medicines, increased uptake of preventive health behaviors, and other behaviors related to health.

## Conclusion

This study aimed to acquire initial insights into the construct validity of the Norwegian adaptation of the HLQ. Despite some possible overlap between two of the scales in part 2 of the questionnaire, the N-HLQ appears relatively robust and might serve as a good foundation for valid measurement in Norwegian populations. However, these findings cannot be generalized to all populations. Future research should include a wider range of respondents, including those in community health, hospital, and home care settings.

## Figures and Tables

**Table 1:** Linkage Between the Nutbeam's Schema of Health Literacy and the Health Literacy Questionnaire

**Nutbeam's schema**	**Broad matching Health Literacy Questionnaire domains**^a^
Basic/functional health literacy: sufficient basic skills in reading and writing to be able function effectively in everyday situations	2. Having sufficient information to manage my health 8. Ability to find good-quality health information 9. Understanding health information well enough to know what to do
Communicative/interactive health literacy: more advanced cognitive and literacy skills that, together with social skills, can be used to actively participate in everyday activities, to extract information and derive meaning from different forms of communication, and to apply new information to changing circumstances	1. Feeling understood and supported by health care providers 3. Actively managing my health 4. Social support for health 6. Ability to actively engage with health care providers 7. Navigating the health system 8. Ability to find good quality-health information
Critical literacy: more advanced cognitive skills, that together with social skills, can be applied to critically analyze information and to use this information to exert greater control over life events and situations	3. Actively managing my health 4. Social support for health 5. Appraisal of health information

Note.

a Within each Health Literacy Questionnaire scale there are some elements of the three levels of Nutbeam's schema, so overlap is expected. Reprinted (permission is not required) under the Creative Commons Attribution License (CC BY) from Osborne et al., 2013.

**Table 2:** Sample Background Characteristics (*N* = 368)

**Characteristic**	***n* (%)**

Age (years)	
Range	18–64
Mean	24
*SD*	7.1

Gender (female)	307 (86)

Speaking Norwegian when growing up	282 (83)

Living with someone	254 (76)

Studying at a university in western Norway	221 (61)

Studying at a Capital university	147 (39)

Long-term health problem or disease	60 (18)

**Table 3:** Internal Consistency of the Nine Health Literacy Questionnaire Scales (Cronbach's Alpha), Mean Scores, and Correlations (x 1,000) Between the Nine Factor Scores and the Predefined Scale Scores (*N* = 368)

**Health Literacy Questionnaire**	**Cronbach's alpha**	**Mean (*SD*)**	**Highest correlation Mean (*SD*)**	**Near highest correlation Mean (*SD*)**	**Difference between the two means**
Health Literacy Questionnaire scales part 1 (possible scores 1–4)					
Feeling understood and supported by health care providers (4 items)	.81	2.96 (0.64)	.936 (11.4)	.413 (58.6)	.523
Having sufficient information to manage my health (4 items)	.76	2.96 (0.47)	.904 (64.2)	.346 (74.5)	.558
Actively managing my health (4 items)	.81	2.86 (0.51)	.950 (30.5)	.328 (81)	.622
Social support for health (4 items)	.78	3.13 (0.51)	.889 (42.3)	.477 (28.2)	.412
Appraisal of health information (5 items)	.80	2.78 (0.49)	.906 (26.4)	.368 (64.6)	.538
Health Literacy Questionnaire scales part 2 (possible scores 1–5)					
Ability to actively engage with health care providers (5 items)	.81	3.56 (0.59)	.899 (40.4)	.451 (82.1)	.448
Navigating in the health care system (6 items)	.75	3.50 (0.56)	772 (51.4)	.546 (90.8)	.226
Ability to find good health information (5 items)	.72	3.69 (0.50)	.686 (60.3)	.529 (58.8)	.157
Understanding health information well enough to know what to do (5 items)	.72	3.71 (0.50)	.860 (71.4)	.470 (55)	.390
